# Study on *LOC426217* as a candidate gene for beak deformity in chicken

**DOI:** 10.1186/s12863-016-0353-x

**Published:** 2016-02-18

**Authors:** Hao Bai, Yanyan Sun, Jing Zhu, Nian Liu, Dongli Li, Fuguang Xue, Yunlei Li, Jilan Chen

**Affiliations:** Key Laboratory of Genetics Resources and Utilization of Livestock, Institute of Animal Science, Chinese Academy of Agricultural Sciences, Beijing, 100193 China

**Keywords:** Beijing-you chickens, Beak deformity, *LOC426217*, SNPs, Haplotype

## Abstract

**Background:**

The beak deformity (crossed beaks) was found in some indigenous chickens of China, such as Beijing-You (BJY), Qingyuan Partridge, and Huxu Chickens. Birds with deformed beaks have reduced feed intake and drinking, impeded growth rate, and poor production performance. Beak deformity reduces the economy of poultry industry and affects animal welfare as well. The genetic basis of this malformation remains incompletely understood. *LOC426217*, also named claw keratin-like, was the most up-regulated gene in the deformed beaks from a previous digital gene expression (DGE) analysis and was selected as an important candidate gene for further analysis.

**Results:**

In the present study, quantitative real-time PCR (qRT-PCR) was firstly performed to determine the expression pattern of *LOC426217* gene in deformed and normal beaks to verify the DGE results. Tissue-specific expression profile of this gene in 14 tissues was also determined using qRT-PCR. The *LOC426217* was amplified from the genomic DNA of 171 deformed and 164 normal beaks, and sequenced to detect the single nucleotide polymorphisms (SNPs). The results showed that *LOC426217* was significantly high-expressed in the deformed beaks, which was in good agreement with the DGE results. This gene was specifically high-expressed in beaks than other tissues. Eight SNPs were detected in *LOC426217*: -62G > T, 24 T > C, 36G > C, 192A > T, 204C > T, 222 T > C, 285G > T, and 363 T > C. Genotype frequency of G-62 T, T24C, G36C, T222C, and T363C loci was significant different between deformed and normal beaks. Haplotype analysis revealed one block with SNPs T24C and G36C, and one block with SNPs A192T, C204T, T222C, and G285T in normal birds, while the block with SNPs G36C and A192T in deformed ones.

**Conclusions:**

It was concluded from these results that the over-expression of *LOC426217* in the beak maybe related to the malformation. The polymorphisms of *LOC426217* gene were associated with the beak deformity trait where the SNPs of G-62 T, T24C, G36C, T222C, and T363C loci maybe used as markers. The specific haplotype block in deformed birds may be a potential linkage marker for this trait.

**Electronic supplementary material:**

The online version of this article (doi:10.1186/s12863-016-0353-x) contains supplementary material, which is available to authorized users.

## Background

The beak is an external structure of birds, consisting of the upper and lower mandibles covered with a thin keratinized layer of epidermis [1]. It is used for many important activities such as feeding, drinking, fighting, and preening. In addition to striking morphological differences between species, beak deformities of different forms (noticeably elongated, crossed, bent at right angles) have been documented in many wild birds [2–7]. Frequencies of 1 % to 3 % of beak deformity (normally a crossed beak) were found in various indigenous chickens of China, such as Beijing-You (BJY) (studied here), Silkies, Qingyuan Partridge, and Huxu Chickens. Chickens with deformed beaks have reduced feed intake and growth rate. Therefore, beak deformity represents an economic as well as an animal welfare problem in poultry industry. According to our observations in a BJY population, in the absence of known environmental factors contributing to the malformation, birds with deformed beaks present consistently in each generation and cannot be eliminated from a population simply on the basis of the phenotype. This indicated the genetic effects underlying this trait. Studies have been performed to identify the teratogenic genes or molecular genetic background of beak deformity. Previously recognized genetic factors associated with beak deformity include some knwon genes such as fibroblast growth factor 8 (*FGF8*) [8], bone morphogenetic protein 4 (*BMP4*) [9–11], calmodulin (*CaM*) [12], and ALX homeobox 1 (*ALX1*) [13]. The over-expression of homeobox A1 (*HOXA1*) and homeobox D3 (*HOXD3*) may result in beak deformity in chicks [14].

**Fig. 1:**
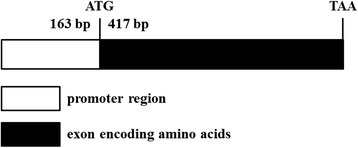
The molecular structure map of *LOC426217* gene. Note: White box: promoter region; black box: exon encoding amino acids; Number: number of base pairs

Sets of differently expressed genes in the deformed and normal beaks have been detected using digital gene expression (DGE) profile analysis based on high-sequencing technology. Of these genes, *LOC426217*, also known as claw keratin-like gene, was the most up-regulated in the deformed beak (log2-Ratio (deformed/normal) = 10.91) [15]. Located on GGA 25, *LOC426217* is a member of the keratin family, containing 417 base pairs with only one exon (Fig. 1). Keratin is crucial for maintaining normal cell morphology involved in the cytoskeleton remodeling keratin filaments and cytoskeletal signaling pathways. Change of its structure results in dysmorphic cells [16]. The cytoskeleton is a complex of intracellular proteins that contribute to shape, support, and movement of cells [17]. Up to now, less study was reported about this gene in chickens. Although highlighted in the DGE analysis, further study of this gene is still needed to verify its roles in beak malformation.

In the present study, qRT-PCR was used to detect the relative expression of *LOC426217* gene in the deformed and normal beaks, to verify the results of DGE profiling. Tissue expression profile of this gene was also determined in 14 tissues of the birds. Eventually, *LOC426217* was amplified and sequenced to seeking the SNPs and haplotypes related with the beak malformation.

## Methods

### Animals and samples collection

The Institutional Animal Care and Use Committee at Institute of Animal Science, Chinese Academy of Agricultural Sciences (IAS, CAAS) approved all procedures involving the use of animals. All efforts were made to minimize the suffering of animals following the animal care guidelines [18]. The animals used in this study came from a pure-line stock of a local breed (Beijing-You) kept by IAS, CAAS (Beijing, China). They were incubated contemporaneously and housed under the same conditions.

The lower mandibles of the beaks were collected from 18 BJY chickens of 56 days of age: 9 with crossed beaks and 9 with normal beaks. Total RNA of the lower mandibles of the crossed and normal beaks above was collected for the verification of DGE profiling results using quantitative real-time PCR (qRT-PCR).

Three normal birds of 56 days of age were killed by stunning and exsanguination. Tissues samples including bursa of fabricius, beak, brain, breast, feather, heart, kidney, thigh, liver, lung, skin, small intestine, stomach, and testicle (50–100 mg) were rapidly collected and snap-froze in liquid nitrogen and storage at -80 °C. The RNA of these samples was used to determine the tissue expression profile of *LOC426217*.

Blood samples were collected from the brachial vein by venipuncture from 171 beak- deformed birds (deformed) and 164 normal ones (control). Based on the case-control study design, we selected these birds according to the phenotype of the birds without family structure. The beak-deformity birds were collected from two generations. The normal birds were selected randomly from the same generation. DNA was isolated from the blood samples and stored at -20 °C for the detection of SNPs located in *LOC426217* gene.

### DNA and RNA extraction and reverse transcription (RT)

Genomic DNA (gDNA) was extracted from blood samples using phenol-chloroform. Total RNA was isolated at 4 °C using the Trizol reagent (Invitrogen, USA). Any residual gDNA and protein were removed with Dnase I (TaKaRa, Japan) and RNA clean kit (TIANGEN, China). The purified RNA was dissolved (200-400 ng/mL, OD260/OD280 = 1.8–2.0), and stored at -80 °C. Total RNA was used for RT (in 20 μL final volume) following the manufacturer's instruction (Promega, USA). The cDNA was stored at -80 °C for subsequent qRT-PCR.

### PCR amplification and qRT-PCR

PCR amplification was performed using PCR Gene Amplifier (Bio-Rad, USA) in a total volume of 25 μL which contains 12 μL of 2 × Taq PCR StarMix (GenStar, China), 1 μL (10 pmol) of each primer (Table 1), 1 μL of gDNA (50 ng) and 10 μL of ddH_2_O. After an initial denaturing for 2 min at 95 °C, there were 35 cycles of amplification (94 °C for 30 s, 60 °C for 30 s, and 72 °C for 90 s) and extension for 5 min at last. PCR products were detected by 1 % agarose gel electrophoresis for 15 min 120 V, stained with ethidium bromide, examined under UV light, and photographed. The PCR products were then sequenced by BGI (Beijing, China).

**Table 1:** Gene-specific primers used in PCR

Gene	Primer sequence	Product length (bp)	Tm (°C)	GenBank No.
*LOC426217*	F: AGTCCTCTATCCAGCTTCCT	806	60	NC_006112.2
	R: GAGTAGGCAGTCAGAGCTTG			

To validate the DGE results, qRT-PCR was performed to determine the expression of *LOC426217* in the 9 deformed and 9 normal beaks using the ABI 7500 Real-time Detection System (Applied Biosystems, USA) and TaKaRa DRR018A reagents. Each 20 μL PCR mixture contained10 μL of SYBR Premix Ex Taq™ II, 0.8 μL (10 pM) of each primer (Table 2), 0.4 μL of ROX Reference Dye II (50×), 2 μL of cDNA (100 ng) and 6 μL of ddH_2_O. After an initial denaturing for 30 s at 95 °C, there were 40 cycles of amplification (95 °C for 5 s and 60 °C for 32 s), followed by thermal denaturing to generate melting curves to verify amplification specificity. *β*-actin was amplified in the same plates as endogenous control. Samples were assayed in triplicate for standard curves. PCR efficiency of the *LOC426217* gene and *β*-actin was consistent. cDNA from normal beaks served as a standard control for tissue-specific expression profile study. The amplification efficiency of transcripts of interest and the internal standard (*β-actin*) were consistent. Dissociation curves verified that amplification was specific.

**Table 2:** Gene-specific primers used in qRT-PCR

Gene	Primer sequence	Product length (bp)	Tm (°C)	GenBank No.
*LOC426217*	F: CACCGTGGTCACCTTCCCCG	157	60	XM_423880
	R: GCCTCCATAGCCACCAAAAC			
*β*-actin	F: GAGAAATTGTGCGTGACATCA	152	60	NM_205518
	R: CCTGAACCTCTCATTGCCA			

### SNPs filter and genotyping

PCR amplification product of *LOC426217* gene was then directly sequenced by BGI company (Beijing, China) using Sanger sequencing methods [19]. The SNPs and amino acids were determined and filtered by the software DNAStar (Version 5.01).

### Statistical analysis

The relative abundance of transcripts was calculated from 2^−ΔΔCT^ [20]. All data presented graphically are means ± SEM. The significance level was *P* < 0.05 or *P* < 0.01. Student’s *t*-tests were used to evaluate the relative expression differences of *LOC426217* between the RNA samples of deformed and normal beaks. The ANOVA procedure of SAS 8.0 was used to assess the differences expression of *LOC426217* in all the tissues. All SNPs were checked for Hardy-Weinberg Equilibrium (HWE) in both groups (*P* > 0.05 means equilibrium). Allele frequency, genotype frequency, and polymorphism information content (*PIC*, *PIC* < 0.25: low polymorphism; 0.25 < *PIC* < 0.5: moderate polymorphism; *PIC* > 0.5: high polymorphism) were calculated by PopGene (Version 1.31). Chi-square tests were used to evaluate the genotype frequency differences between deformed and normal beaks. Benjamini & Hochberg method was used for the Bonferroni correction [21]. Linkage Disequilibrium (LD) pattern for the SNPs genotyped was plotted using Haploview (Version 4.2). The sliding window method was used to generate different haplotypes between two groups [22, 23].

## Results

### Verification for *LOC426217* gene of DGE results

**Fig. 2:**
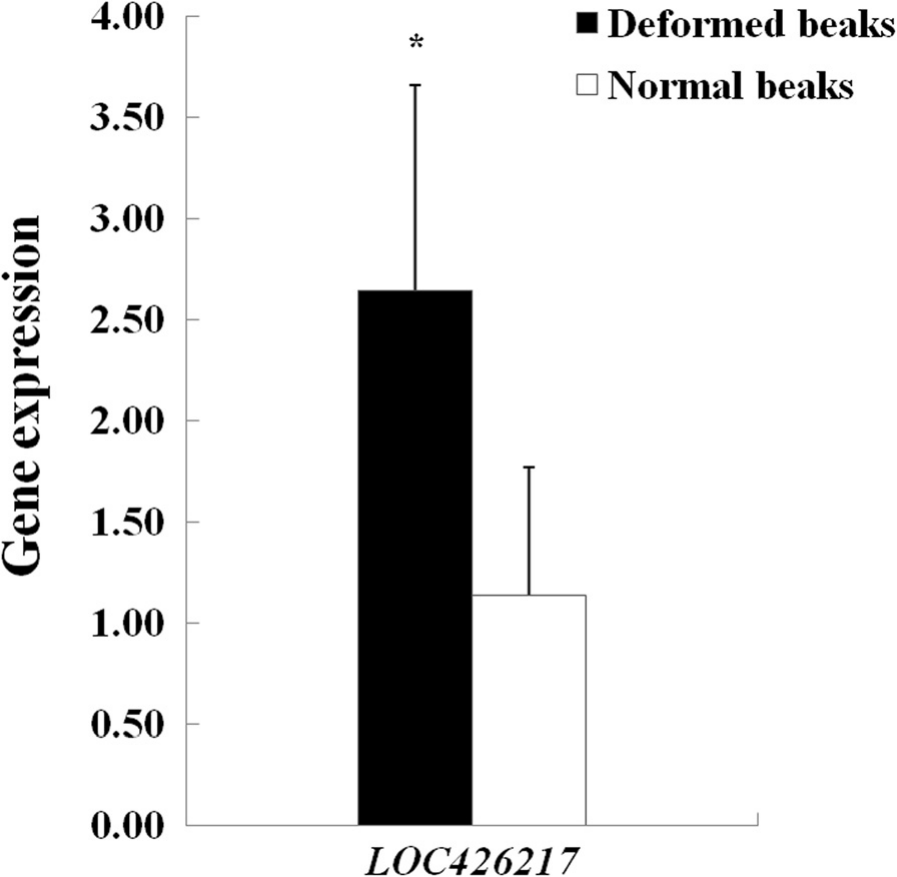
Relative expression of *LOC426217* in 9 deformed and 9 normal beaks. Note: * means significant difference between two groups (*P* < 0.05)

To verify the previous DGE analysis, where *LOC426217* was the most up-regulated in the deformed beaks, qRT-PCR was used to estimate the expression of this gene in 9 deformed and 9 normal beaks. As shown in Fig. 2, the relative expression of *LOC426217* in deformed beaks was significantly higher than that in normal ones (*P* < 0.05). This was in good agreement with the DGE analysis.

### Tissue expression profile of *LOC426217*

**Fig. 3:**
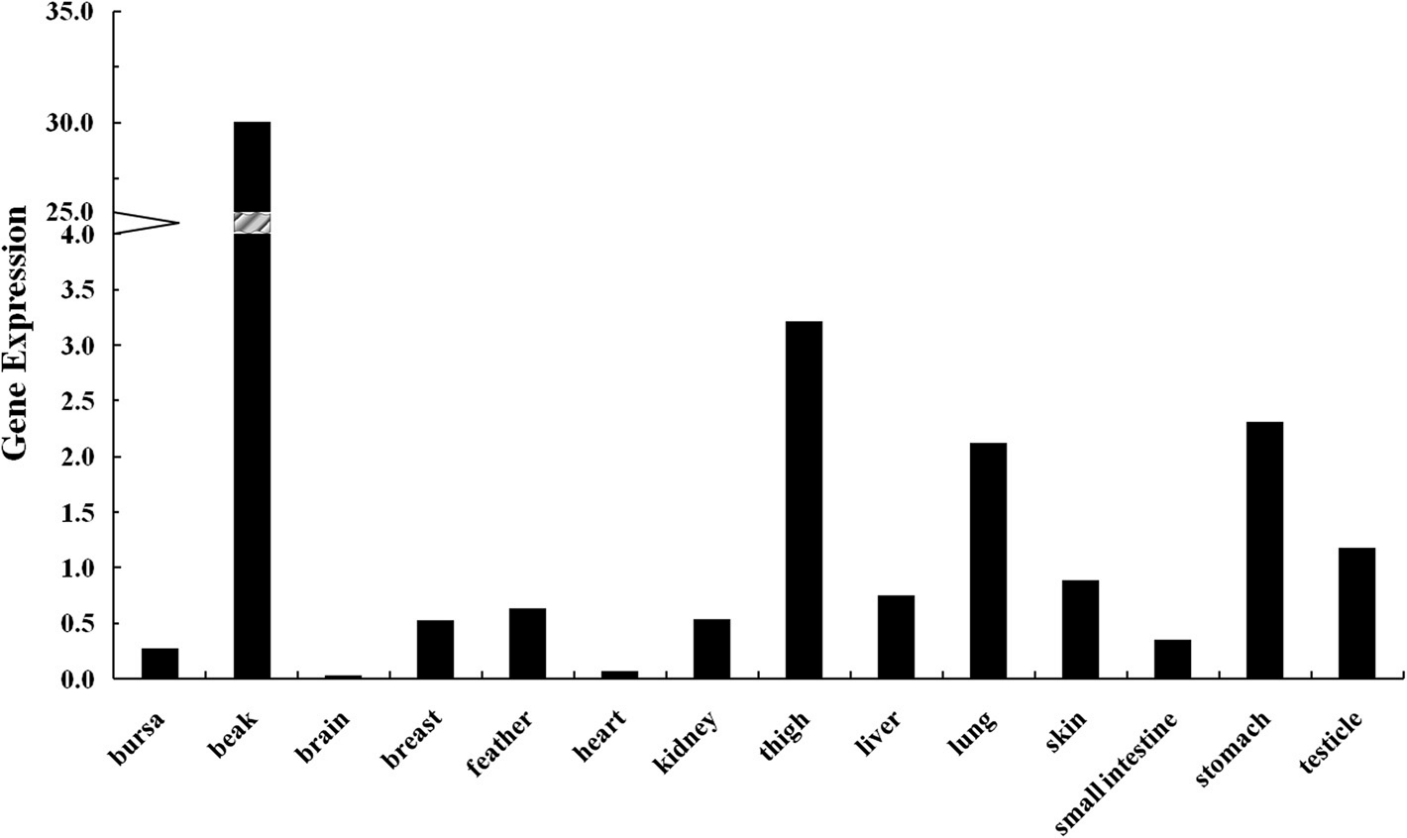
Relative expression of *LOC426217* gene in 14 tissues of the chicken

As shown in Fig. 3, *LOC426217* gene was hardly expressed in brain, heart, bursa, or small intestine. The relative expression in beak was significantly higher than that in other tissues (*P* < 0.01). The results revealed that this gene maybe specifically expressed in beak tissue. In order to seek the structural changes of this gene, DNA sequencing was carried out subsequently.

### DNA sequencing and SNPs detection

**Fig. 4:**
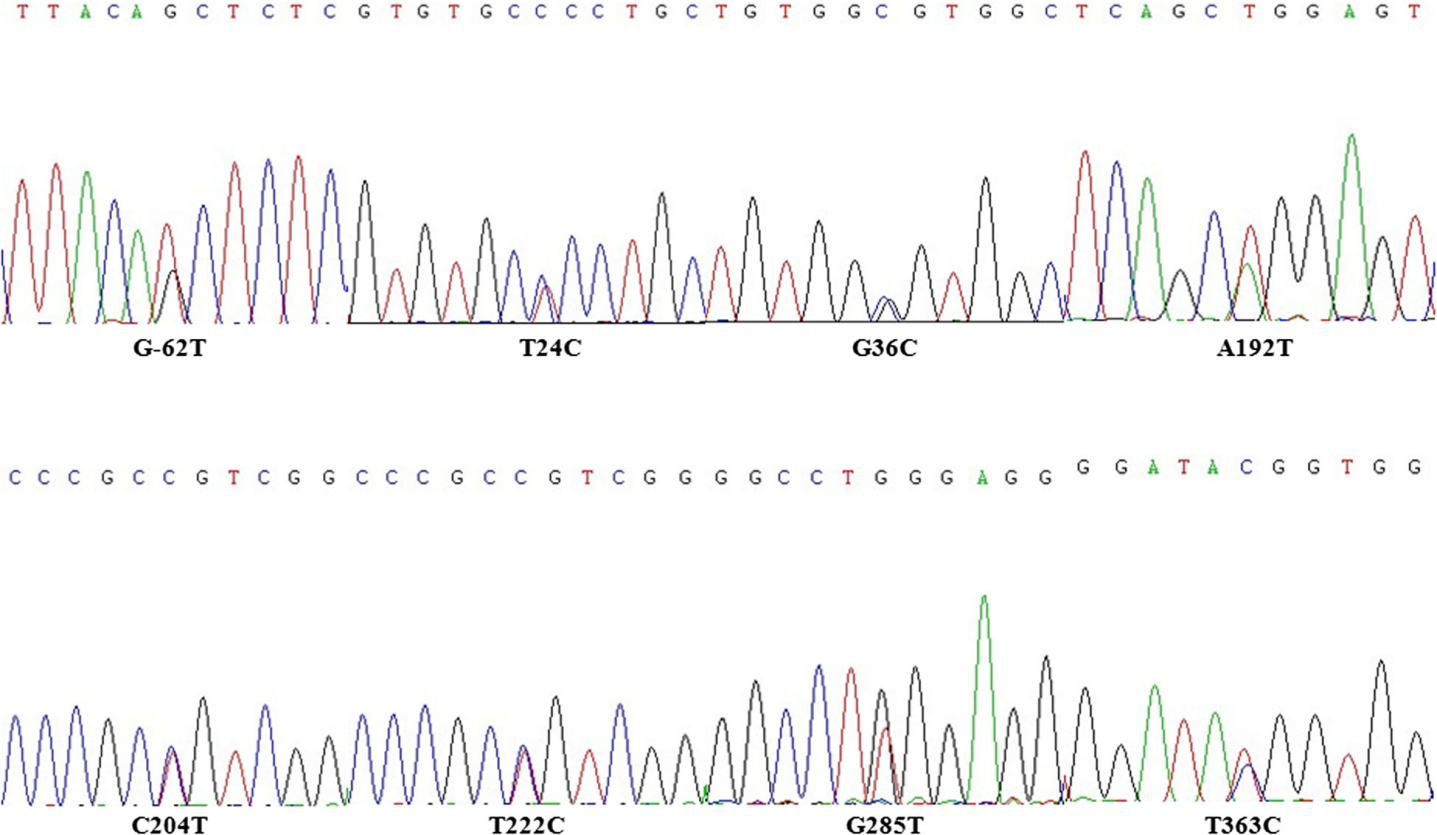
Sequencing map of the mutation loci in *LOC426217* in chicken

*LOC426217* gene of 171 beak-deformed and 164 normal birds was amplified and sequenced. Eight SNPs were detected, including one locus in promoter region: -62G > T, and seven loci in coding region: 24 T > C, 36G > C, 192A > T, 204C > T, 222 T > C, 285G > T, and 363 T > C (Fig. 4). The loci in coding region were synonymous mutations resulting with no amino acid changing. These SNPs were presented in both beak-deformed and normal birds. The 18 birds used in the qRT-PCR analysis for DGE verification were also sequenced and their genotypes were shown in Table 3 and Additional file 1: Table S1.

**Table 3:** Genotypes of the birds used for qRT-PCR analysis for validation of Digital Gene Expression results

Trait	No.	SNP Loci							
		G-62 T	T24C	G36C	A192T	C204T	T222C	G285T	T363C
Deformed	1	TT	TC	GC	AT	CT	TT	GT	CC
	2	TT	TC	GC	AT	CT	TT	GT	TT
	3	TT	CC	CC	AA	CT	TC	GG	TT
	4	GT	TC	GC	AA	CC	TC	GG	TT
	5	GT	TC	GC	AA	CC	TC	GG	TT
	6	TT	TT	GG	AA	TT	TT	GG	TT
	7	TT	TT	GG	AT	CC	TT	GT	CC
	8	TT	TT	GG	AT	CC	TT	GT	TT
	9	GT	TC	GC	AT	CC	TC	GT	TT
Normal	10	TT	TC	GC	AT	CC	TC	GT	TT
	11	TT	CC	CC	AA	TT	TT	GG	TT
	12	TT	TC	GC	AA	CT	TC	GG	TT
	13	TT	TC	GC	AA	TT	TT	GG	TT
	14	TT	TC	GC	AT	CT	TT	GT	CC
	15	TT	TC	GC	AT	CT	TT	GT	CC
	16	TT	TC	GC	AA	CT	TC	GG	TT
	17	TT	CC	CC	AA	CT	TC	GG	TT
	18	TT	TC	GC	AT	CC	TC	GT	TT

### Genetic diversity analysis

Genetic diversity was analyzed by PopGene (Version 1.31). All SNPs were firstly checked for HWE. In the normal birds (n = 164), T24C, G36C, A192T, T222C, G285T, and T363C were not in agreement with the HWE (*P* < 0.05), while in the birds with a deformed beak (n = 171), T24C, G285T, and T363C were not in agreement with the HWE. Two loci (G-62 T and C204T) of normal beaks and two loci (G-62 T and T363C) of deformed ones were low polymorphism (*PIC* < 0.25) (Tables 4 and 5).

**Table 4:** Genetic diversity analysis of *LOC426217* in normal birds

Loci	Genotype	Genotype frequency (n)	Allele	Allele frequency	*χ* ^*2*^ value (*P* value)	*PIC*
G-62 T	GG	0.00(0)	G T	0.13 0.87	3.837 (0.050)	0.205
	GT	0.27 (44)				
	TT	0.73 (120)				
T24C	TT	0.36 (59)	T C	0.65 0.35	10.32 (0.001)*	0.353
	TC	0.57 (94)				
	CC	0.07 (11)				
G36C	GG	0.48 (78)	G C	0.72 0.28	5.48(0.019)*	0.324
	GC	0.48 (79)				
	CC	0.04 (7)				
A192T	AA	0.53 (86)	A T	0.76 0.24	10.58 (0.001)*	0.301
	AT	0.46 (76)				
	TT	0.01 (2)				
C204T	CC	0.70 (115)	C T	0.83 0.17	0.13 (0.721)	0.237
	CT	0.27 (44)				
	TT	0.03 (5)				
T222C	TT	0.55 (90)	T C	0.76 0.24	6.71(0.0096)*	0.295
	TC	0.43(71)				
	CC	0.02 (3)				
G285T	GG	0.54(89)	G T	0.76 0.24	9.17 (0.0025)*	0.295
	GT	0.45 (73)				
	TT	0.01(2)				
T363C	TT	0.70(115)	T C	0.79 0.21	36.58 (<0.0001)*	0.277
	TC	0.18 (29)				
	CC	0.12(20)				

Note: *χ* ^*2*^ value means the test values of different genotypes to Hardy-Weinberg equilibrium. * (*P* < 0.05) means the loci were not in agreement with the Hardy-Weinberg equilibrium

**Table 5:** Genetic diversity analysis of *LOC426217* in beak-deformed birds

Loci	Genotype	Genotype frequency (n)	Allele	Allele frequency	*χ* ^*2*^ value (*P* value)	*PIC*
G-62 T	GG	0.02 (3)	G T	0.10 0.90	1.37 (0.242)	0.163
	GT	0.16(28)				
	TT	0.82 (140)				
T24C	TT	0.24(41)	T C	0.535 0.465	5.80 (0.016)*	0.374
	TC	0.59(101)				
	CC	0.17 (29)				
G36C	GG	0.32 (54)	G C	0.594 0.406	3.77 (0.050)	0.366
	GC	0.55(95)				
	CC	0.13 (22)				
A192T	AA	0.56(96)	A T	0.760 0.240	1.33 (0.249)	0.298
	AT	0.40 (68)				
	TT	0.04(7)				
C204T	CC	0.59(101)	C T	0.781 0.219	1.98 (0.160)	0.284
	CT	0.38(65)				
	TT	0.03 (5)				
T222C	TT	0.50 (85)	T C	0.699 0.301	0.34 (1.562)	0.332
	TC	0.40(69)				
	CC	0.10 (17)				
G285T	GG	0.57 (97)	G T	0.772 0.228	4.38 (0.036)*	0.290
	GT	0.41(70)				
	TT	0.02(4)				
T363C	TT	0.82 (140)	T C	0.848 0.152	104.165 (<0.0001)*	0.225
	TC	0.06 (10)				
	CC	0.12(21)				

Note: *χ* ^*2*^ value means the test values of different genotypes to Hardy-Weinberg equilibrium.* (*P* < 0.05) means the loci were not in agreement with the Hardy-Weinberg equilibrium

### Genotype frequency differences in the beak-deformed and normal birds

Chi-square tests were used to evaluate the genotype frequency differences of SNP loci in *LOC426217* gene between two groups with a Bonferroni correction for the *P*-values. As shown in Table 6, the genotype frequency of G-62 T locus showed significant difference between two groups (*P* < 0.05), while the genotype frequencies of T24C, G36C, T222C and T363C loci showed highly significant differences (*P* < 0.01). There was no significant difference for the rest SNPs (*P* > 0.05).

**Table 6:** Genotypes frequency comparison of all the loci between beak-deformed and normal birds

Loci	Trait	Genotype			*χ* ^*2*^ value	*P-*value	Corrected *P-*value
		AA	AB	BB			
G-62 T	normal deformity	0	44	120	7.951	0.019	0.030*
		3	28	140			
T24C	normal deformity	59	94	11	11.450	0.003	0.008**
		41	101	29			
G36C	normal deformity	78	79	7	13.453	0.001	0.008**
		54	95	22			
A192T	normal deformity	86	76	2	3.627	0.163	0.186
		96	68	7			
C204T	normal deformity	115	44	5	4.809	0.090	0.120
		101	65	5			
T222C	normal deformity	90	71	3	9.829	0.007	0.014*
		85	69	17			
G285T	normal deformity	89	73	2	0.928	0.629	0.629
		97	70	4			
T363C	normal deformity	115	29	20	11.591	0.003	0.008**
		140	10	21			

Note: *χ* ^*2*^ value means the tested values of different genotypes between two groups. * means significant difference between two groups (*P* < 0.05). ** means highly significant difference between two groups (*P* < 0.01). Corrected *P*-value using Benjamini & Hochberg method [21] was used for the Bonferroni correction

### Haplotype analysis

**Fig. 5:**
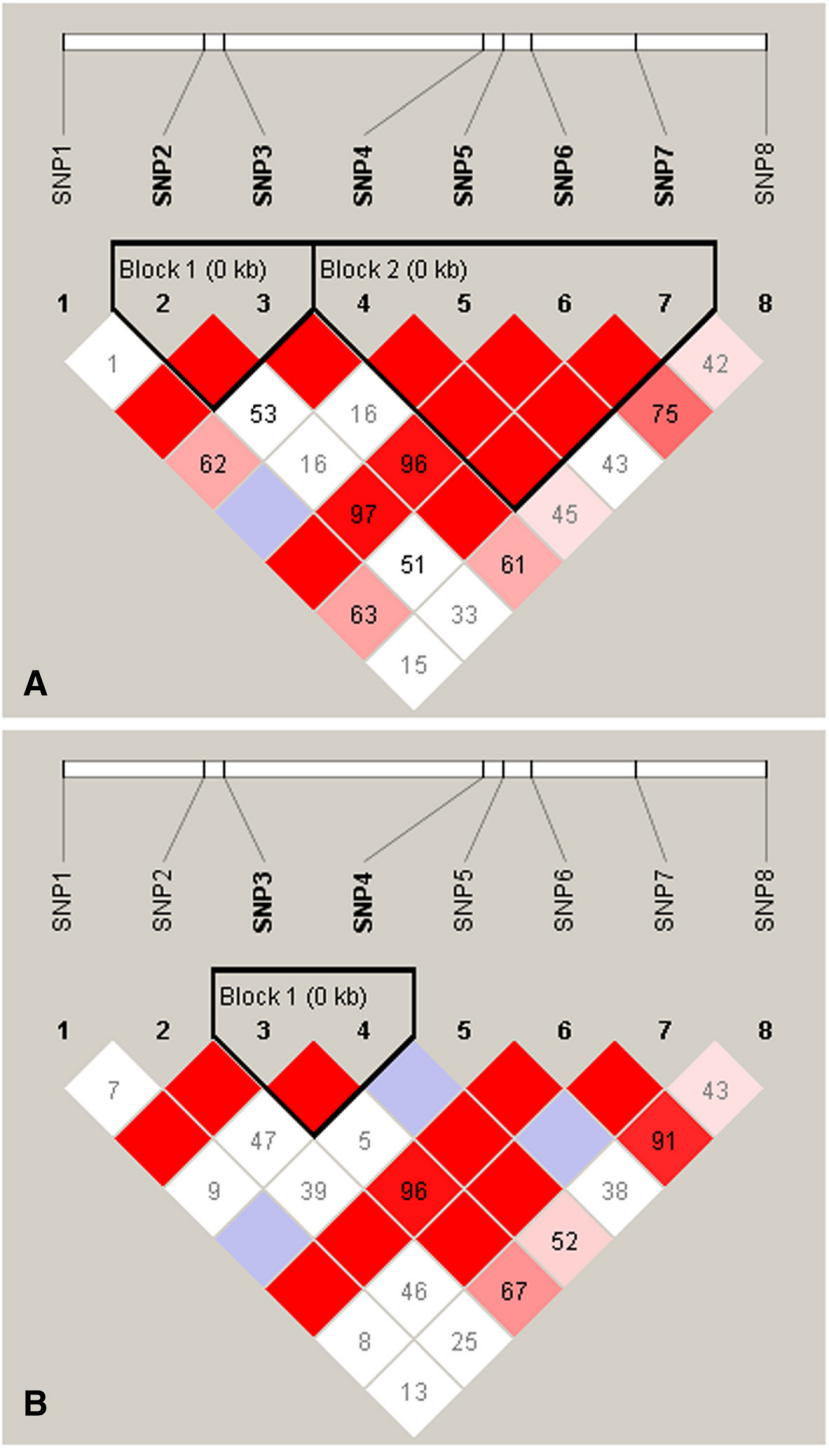
Haplotype blocks of *LOC426217* in the normal (**a**) and deformed (**b**) beaks birds. SNP1: G-62 T; SNP2: T24C; SNP3: G36C; SNP4: A192T; SNP5: C204T; SNP6: T222C; SNP7: G285T; SNP8: T363C. In the normal birds, two haplotype blocks were identified: one block with SNPs T24C and G36C, and one block with SNPs A192T, C204T, T222C, and G285T. In the birds with a deformed beak, one block with SNP G36C and A192T was identified

In the normal birds, two haplotype blocks of *LOC426217* were identified: one block with SNPs T24C and G36C, and one block with SNPs A192T, C204T, T222C, and G285T. In the birds with a deformed beak, one block with SNP G36C and A192T was identified (Table 7 and Fig. 5). The haplotype blocks of deformed birds were different from those of the normal ones.

**Table 7:** Haplotype frequencies in the normal- and deformed-beaks birds

Normal				Deformed	
Block1		Block2		Block	
Haplotype	Frequency	Haplotype	Frequency	Haplotype	Frequency
TG	0.646	ACTG	0.357	CA	0.406
CC	0.284	ACCG	0.235	GA	0.354
CG	0.070	TCTT	0.235	GT	0.240
		ATTG	0.165

## Discussion

The molecular genetic mechanism underlying beak deformity is likely to be very complex. Beak deformities of different forms (noticeably elongated, crossed, bent at right angles) have been documented in many wild birds. The molecular mechanism of beak deformity trait is not clear yet. For the wild birds, it is difficult to obtain the individuals for genetic study. Beak deformity was also found in various indigenous chickens and it is easy to collect individuals. This made the chicken a perfect model for the genetic study of this defect. Based on the previous DGE profiling and bioinformatics analyses, we identified a cluster of differentially expressed genes (DEGs) in the deformed and normal beaks. Some of the DEGs were quite extreme, especially the *LOC426217* gene in the present study.

### Validation and tissue expression profile

In order to validate the reliability and accuracy of DGE results, qRT-PCR was performed. The results showed that the expression of *LOC426217* in the deformed beaks was significantly higher than that in the normal beaks, which was in great agreement with DGE analysis. The result of tissue expression profile also revealed that this gene was specifically expressed in beak tissue. *LOC426217* is a member of the keratin family [24]. Keratin is a key gene family for maintaining normal cell morphology [16]. Variation of keratin structure can lead to beak deformity [25]. It is also an intermediate filament protein that has essential functions in maintaining the structural integrity of epidermis and its appendages [26], presumably including the beak. In addition, keratin is the main composition of the chicken beak. This may be the main reason for its high expression in beak tissue. According to our early observation of beak anatomy, the lower mandibles of the beaks were abnormal/asymmetry. It was reported that avian keratin disorder could result in gross over growth of the rhamphotheca [7]. The beak deformity caused by the excessive growth of one side of the lower mandibles maybe a result of abnormal high expression of *LOC426217*.

### SNPs and haplotypes associated with beak deformity

Based on the case-control study design, the investigated birds used for sequencing were selected from the pure line BJY population according to the beak phenotype of the birds with no family structures. Since the percentage of beak-deformity birds in the chicken population was 1 %-3 %, it was not easy to collect enough birds for gene sequencing. The beak-deformity birds were collected from two generations (about 5000 birds in each generation) and the normal birds were selected randomly from the same generation.

Using direct sequencing, eight SNPs located in the *LOC426217* gene were detected, including one locus in promoter region and seven loci in coding region. These loci were found in both birds with deformed and normal beaks. Four loci (T24C, G36C, T222C, and T363C) were previously released on the database of NCBI (http://www.ncbi.nlm.nih.gov) while the rest four SNPs found in this study were new. Furthermore, five SNPs, individuals with different genotypes showed significant differences between beak-deformed and normal birds (*P* < 0.05). Previous research demonstrated that promoter mutation is a kind of the mutations which can enhance or reduce the expression level of a gene and lead to different phenotypes [27, 28]. In this current study, one SNP (-G62T) was detected in the promoter region and the genotype frequency was significant different between two groups. This mutation could possibly affect the start, expression time and level of this gene. As shown in Table 3, the genotype of 9 normal birds were all TT, while 3 out of the 9 beak-deformed were TG. Further validation study with larger sample size is needed to validate the mRNA expression profile of different genotypes. Similarly, the mutations in coding region are also very important. As we all know, exon is a nucleotide sequence in DNA that carries the code for the final mRNA molecule and thus defines the amino acid sequence during protein synthesis. If the base pairs changed in coding region, the structure and function of protein could possibly change, such as missense mutation or silent mutation [29–32]. In this study, all the loci in coding region were synonymous mutations with no amino acid and protein changing. Synonymous mutations, which do not alter the protein sequence, have also been shown to affect protein function [33, 34] and play key roles in human diseases [35]. Therefore, the mutations detected here may also affect this gene function. Sometimes, the phenotype altering or diseases occurring was not associated with one single SNP changing but related to several SNPs. Global patterns of DNA sequence variation (haplotypes) defined by common SNPs have important implications for identifying disease and traits [36, 37]. A previous study showed that the *ALX1* haplotype has contributed to diversification of beak shapes among the Darwin’s finches and, thereby, to an expanded utilization of food resources [13]. In this present study, different haplotypes were analyzed between two groups. It indicated that the beak deformity trait might be related to the specific haplotype block which was only existed in beak-deformed chickens. To sum up, SNPs and haplotypes described here were interesting and worthy of further study.

## Conclusions

To the best of our knowledge, this is the first time that *LOC426217* was studied as an important candidate gene for beak deformity in birds. The over-expression of *LOC426217* may be the cause of beaks malformation. The genotype frequency of SNPs at G-62 T, T24C, G36C, T222C, and T363C loci showed significant differences between deformed and normal birds, and might be used as candidate SNP markers for this trait. The specific haplotype block in the deformed group could be served as a potential linkage marker for this trait. Further functional verification studies like over-expression or RNA interfere of *LOC426217* during the embryonic development are required to reveal its roles in the beak malformation.

### Availability of supporting data

All the supporting data are included in the manuscript as well as additional files in the supplementary section.

## Additional files

Additional file 1: Table S1. Genotypes of all the 171 beak-deformed and 164 normal birds in this study (XLSX 26 kb) Additional file 2:
**The ARRIVE Guidelines Checklist Animal Research: Reporting In Vivo Experiments.** (DOCX 661 kb)

## Abbreviations

*ALX1*: ALX homeobox 1
BJY: Beijing-You
*BMP4*: Bone morphogenetic protein 4
*CaM*: Calmodulin
DEGs: Differentially expressed genes
DGE: Digital gene expression
*FGF8*: Fibroblast growth factor 8
gDNA: Genomic DNA
*HOXA1*: Homeobox A1
*HOXD3*: Homeobox D3
HWE: Hardy-Weinberg Equilibrium
LD: Linkage Disequilibrium
*PIC*: Polymorphism information content
qRT-PCR: Quantitative real-time PCR
RT: Reverse transcription
SNPs: Single nucleotide polymorphisms
