# Supplementary material for: Study on LOC426217 as a candidate gene for beak deformity in chicken
Source: BMC Genet. 2016 Feb 18;17:44. doi: 10.1186/s12863-016-0353-x (PMC4758156; doi:10.1186/s12863-016-0353-x)

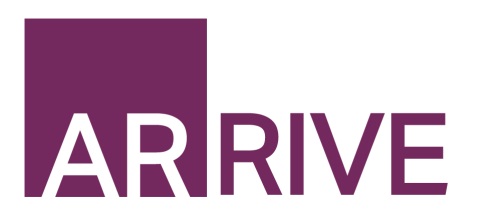


The ARRIVE Guidelines Checklist

Animal Research: Reporting In Vivo Experiments

Carol Kilkenny^1^, William J Browne^2^, Innes C Cuthill^3^, Michael Emerson^4^ and Douglas G Altman^5^

*^1^The National Centre for the Replacement, Refinement and Reduction of Animals in Research, London, UK, ^2^School of Veterinary Science, University of Bristol, Bristol, UK, ^3^School of Biological Sciences, University of Bristol, Bristol, UK, ^4^National Heart and Lung Institute, Imperial College London, UK, ^5^Centre for Statistics in Medicine, University of Oxford, Oxford, UK.*

|  | | ITEM | RECOMMENDATION | Section/ Paragraph |
| --- | --- | --- | --- | --- |
| 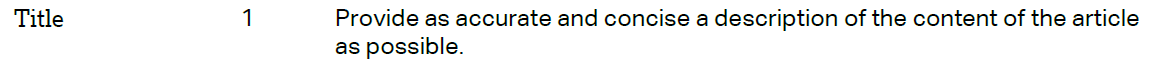 | | | **Title** |  |
| 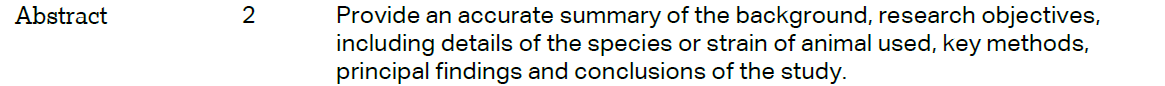 | | | **Abstract** |  |
| INTRODUCTION | | |  |  |
| 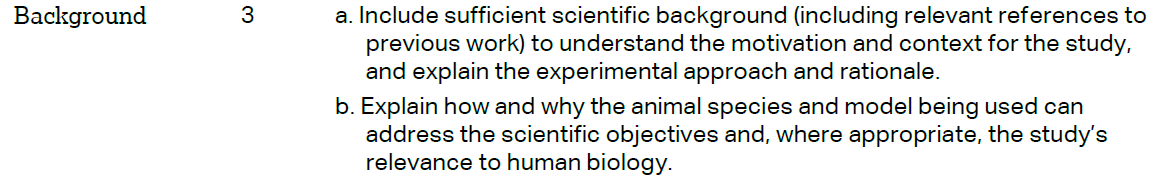 | | | **Paragraphs 1-2**  **Paragraphs 2-3** |  |
| 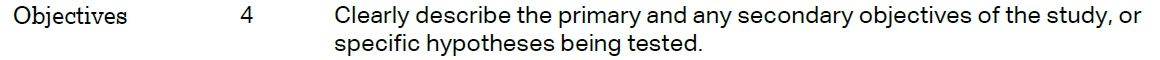 | | | **Paragraph 3** |  |
| METHODS | | |  |  |
| 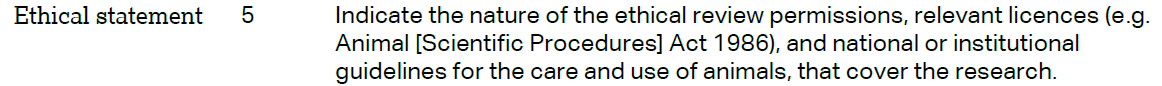 | | | **Paragraph 1** |  |
| 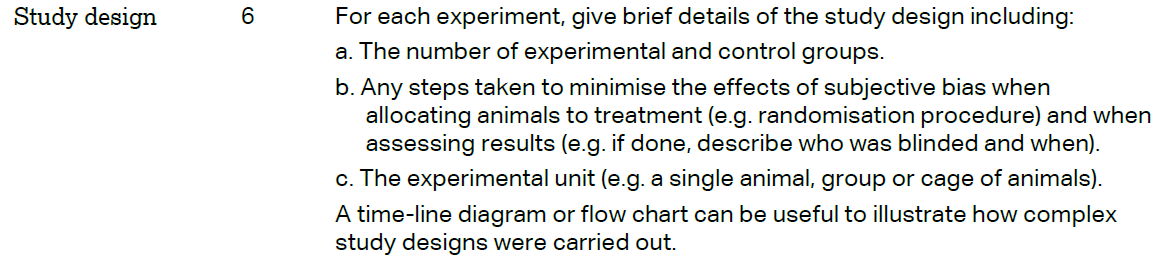 | | | **Paragraphs 2-4** |  |
| 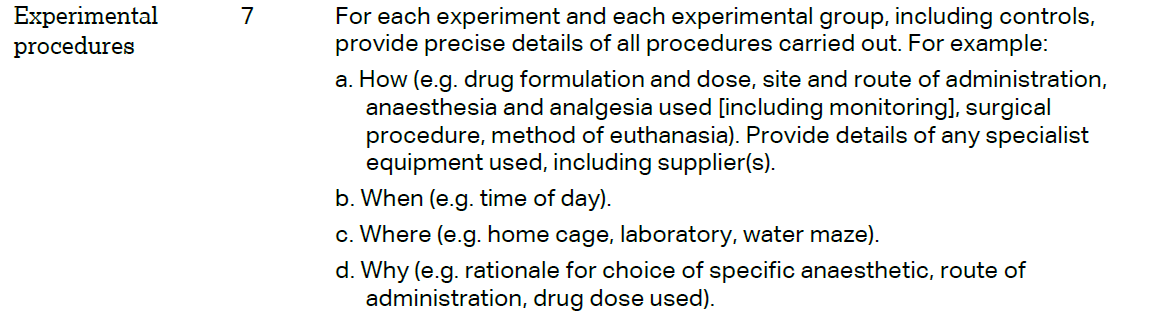 | | | **Paragraphs 5-8** |  |
| 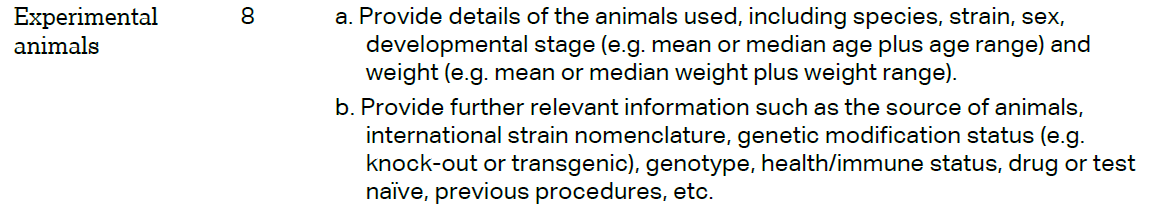 | | | **Paragraph 1** |  |

The ARRIVE guidelines. Originally published in *PLoS Biology*, June 2010^1^

| 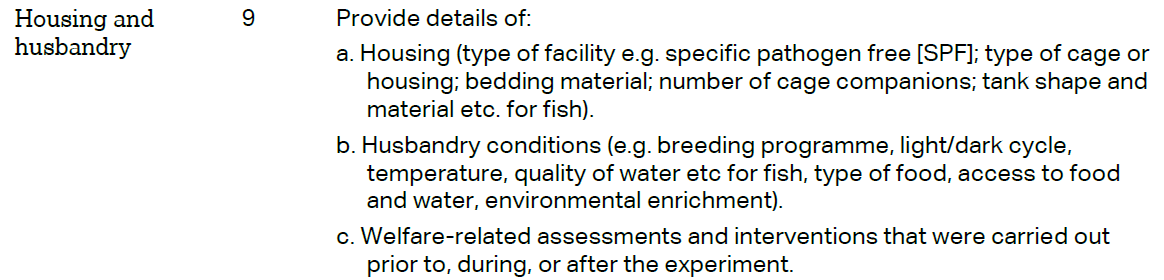 | **Paragraph 1** |  |
| --- | --- | --- |
| 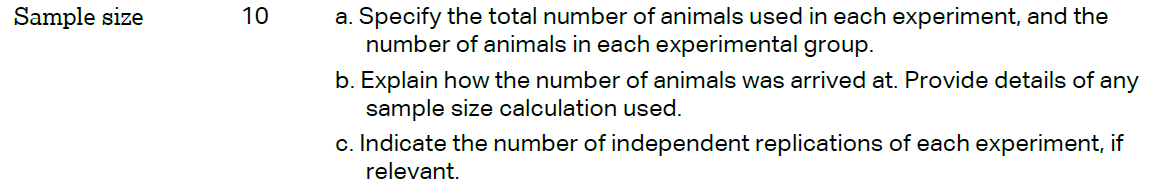 | **Paragraphs 2-4** |  |
| 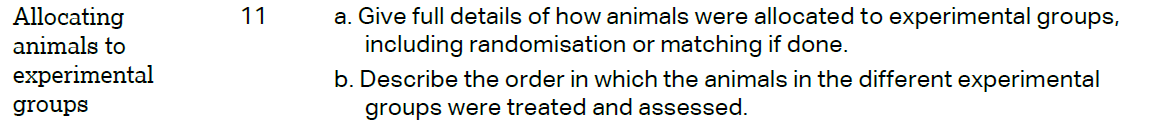 | **Paragraphs 2-4** |  |
| 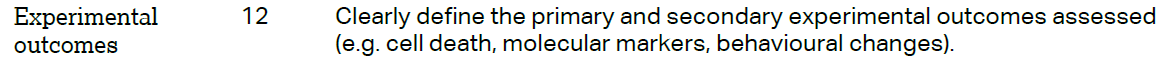 | **Paragraph 9** |  |
| 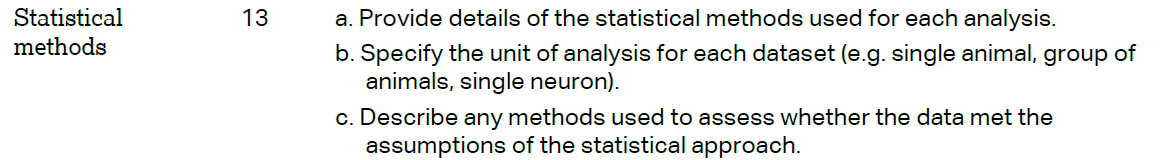 | **Paragraph 9** |  |
| RESULTS |  |  |
| 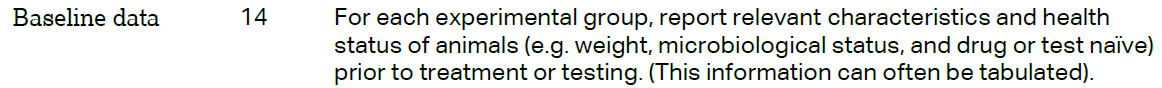 | **Methods**  **paragraph 1** |  |
| 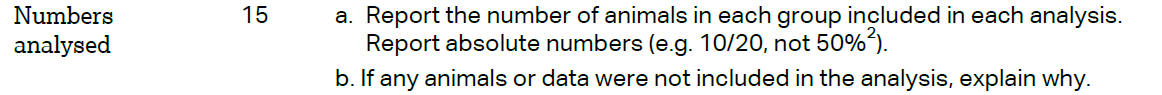 | **Methods**  **paragraphs 2-4** |  |
| 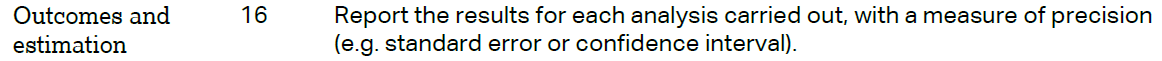 | **Paragraphs 1-6** |  |
| 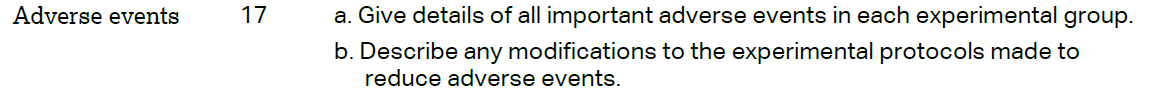 | **NA** |  |
| DISCUSSION |  |  |
| 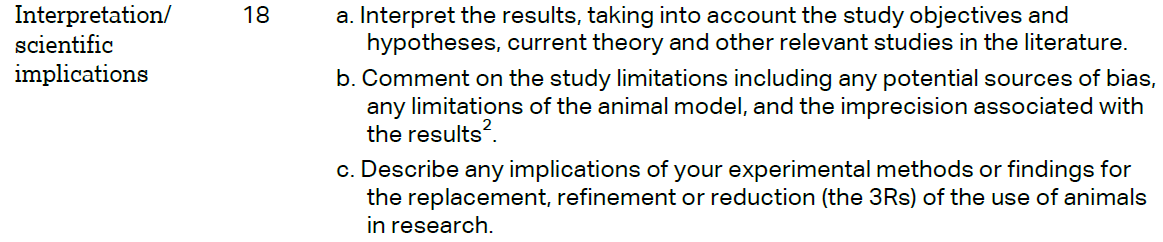 | **Throughout**  **Paragraphs**  **2 & 4** |  |
| 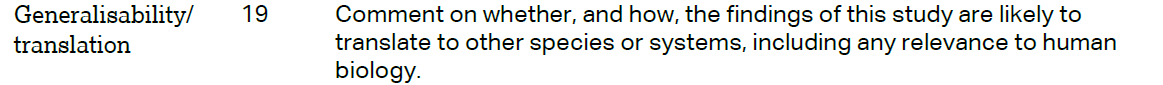 | **Paragraph 1** |  |
| 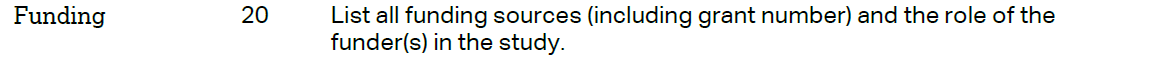 | | **Acknowledgements section** |


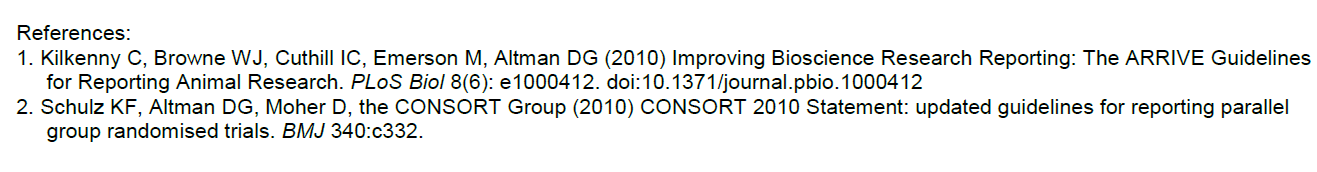

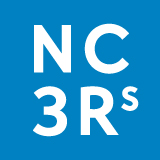

Supplement: Additional file 2: — The ARRIVE Guidelines Checklist Animal Research: Reporting In Vivo Experiments. (DOCX 661 kb) [file 12863_2016_353_MOESM2_ESM.docx]
